# Existing Drugs Considered as Promising in COVID-19 Therapy

**DOI:** 10.3390/ijms22115434

**Published:** 2021-05-21

**Authors:** Edyta Janik, Marcin Niemcewicz, Marcin Podogrocki, Joanna Saluk-Bijak, Michal Bijak

**Affiliations:** 1 Biohazard Prevention Centre, Faculty of Biology and Environmental Protection, University of Lodz, Pomorska 141/143, 90-236 Lodz, Poland; edyta.janik@unilodz.eu (E.J.); marcin.niemcewicz@biol.uni.lodz.pl (M.N.); marcin.podogrocki@biol.uni.lodz.pl (M.P.); 2 Department of General Biochemistry, Faculty of Biology and Environmental Protection, University of Lodz, Pomorska 141/143, 90-236 Lodz, Poland; joanna.saluk@biol.uni.lodz.pl

**Keywords:** COVID-19, SARS-CoV-2, therapy, molecular aspects, drugs

## Abstract

COVID-19 is a respiratory disease caused by newly discovered severe acute respiratory syndrome coronavirus 2 (SARS-CoV-2). The disease at first was identified in the city of Wuhan, China in December 2019. Being a human infectious disease, it causes high fever, cough, breathing problems. In some cases it can be fatal, especially in people with comorbidities like heart or kidney problems and diabetes. The current COVID-19 treatment is based on symptomatic therapy, so finding an appropriate drug against COVID-19 remains an immediate and crucial target for the global scientific community. Two main processes are thought to be responsible for the COVID-19 pathogenesis. In the early stages of infection, disease is determined mainly by virus replication. In the later stages of infection, by an excessive immune/inflammatory response, leading to tissue damage. Therefore, the main treatment options are antiviral and immunomodulatory/anti-inflammatory agents. Many clinical trials have been conducted concerning the use of various drugs in COVID-19 therapy, and many are still ongoing. The majority of trials examine drug reposition (repurposing), which seems to be a good and effective option. Many drugs have been repurposed in COVID-19 therapy including remdesivir, favipiravir, tocilizumab and baricitinib. The aim of this review is to highlight (based on existing and accessible clinical evidence on ongoing trials) the current and available promising drugs for COVID-19 and outline their characteristics.

## 1. Introduction

Coronavirus Disease 2019 known as COVID-19 is caused by a novel severe acute respiratory syndrome coronavirus 2 (SARS-CoV-2) and identified in December 2019, in the city of Wuhan, Hubei Province, China [1]. In March 2020, the World Health Organization (WHO) announced a COVID-19 outbreak as a pandemic. For more than a year, the world has been facing the COVID-19. According to the WHO report, in early March 2021, over 117 million confirmed cases of COVID-19 were reported, including more than 2,600,000 deaths [2].

Like other coronaviruses, SARS-CoV-2 particles are enveloped, spherical shaped containing genetic material inside. Spike (S) proteins play an essential role in the process of viral invasion. A large number of S proteins cover the virus surface and bind to angiotensin-converting enzyme 2 (ACE2) host cell receptor, mediating viral cell entry [3]. While S protein binds to the receptor, Transmembrane protease serine 2 (TMPRSS2), which is a type 2 transmembrane serine protease located on the membrane of the host cell, promotes virus entry into the cell. When the virus enters host cell, viral RNA is released. The polyproteins are translated from RNA genome, and the viral RNA genome is replicated and transcribed by cleaving the protein and assembling the replicase–transcriptase complex. Viral RNA is replicated, and structural proteins are synthesized, assembled, and packaged in the host cell. Furthermore, viral particles are released from infected host cell [4].

**Figure 1:**
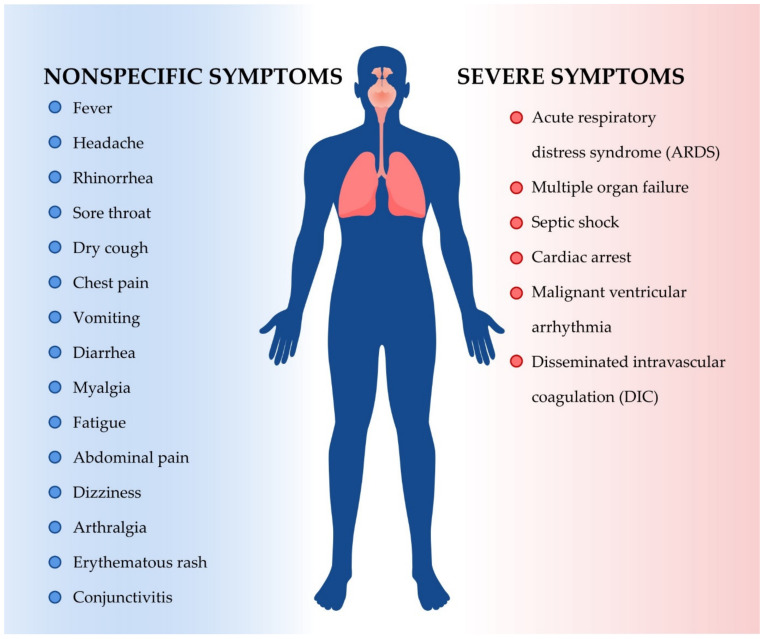
The range of SARS-CoV-2 infection symptoms. The symptoms of SARS-CoV-2 infection are both nonspecific and severe. Among the nonspecific symptoms, which can also be symptoms of a cold or flu, fever, headache, rhinorrhea, dry cough, chest pain, fatigue, dizziness and conjunctivitis can be distinguished. Severe symptoms include serious lung condition (acute respiratory distress syndrome), cardiological problems (cardiac arrest, malignant ventricular arrhythmia) and different serious disorders like multiple organ failure, septic shock and disseminated intravascular coagulation. ARDS: acute respiratory distress syndrome; DIC: disseminated intravascular coagulation.

The transmission among people occurs through respiratory droplets, which are released when an infected person coughs, sneezes or speaks [5]. SARS-CoV-2 causes respiratory tract infections and clinical spectrum ranges from asymptomatic and nonspecific infection to critical conditions like acute respiratory distress syndrome (ARDS) (Figure 1) [6]. Symptoms in adults usually appear 2–14 days after exposure. Manifestations usually begin with nonspecific flu-like symptoms like dry cough, fever, myalgia or fatigue [7]. Various systems may be involved, including respiratory (cough, short of breath, rhinorrhea, sore throat, hemoptysis, and chest pain), musculoskeletal (muscle ache), gastrointestinal (diarrhea, abdominal pain and vomiting), olfactory (hyposmia, anosmia or complete loss of olfactory functions), ophthalmic (conjunctivitis, retinitis), dermatological (erythematous rash, chickenpox-like vesicles), cardiovascular (arrhythmias), rheumatological (arthralgia) and neurologic (headache and confusion) [8,9,10]. In case of pediatric patients, the disease incubation period in comparison to adults is a little bit longer up to 14 days [11]. However, disease course is mainly asymptomatic or mild [12]. Although, in April 2020 Pediatric Inflammatory Multisystem Syndrome Temporally (PIMS-TS) associated with COVID-19 infection among children manifested by symptoms of Kawasaki disease, Toxic Shock Syndrome (TSS) and viral sepsis was firstly observed. The syndrome is very disturbing and requires multidisciplinary setting, teams (pediatricians, cardiologists, and hematologists) and further research in order to provide adequate treatment options [13].

In adults, according to the study of 99 COVID-19 patients with mean age of 55 and mostly men 68%, the disease in laboratory tests manifests by an increase in the total number of C-reactive protein (CRP), Interluekin-6 (IL-6), neutrophils, and leucocytes [14]. Lymphocytes including CD4+ and CD8+ T cells, B cells and natural killer (NK) cells were remarkably decreased [15]. Patients with severe COVID-19 symptoms demonstrate high levels of Interluekin-2 (IL-2), Interluekin-7 (IL-7), Interluekin-10 (IL-10), granulocyte colony-stimulating factor (G-CSF), tumor necrosis factor-α (TNF-α), IFN-gamma-inducible protein 10 (IP-10), monocyte chemoattractant protein 1 (MCP1), and macrophage inflammatory protein 1α (MIP1α) in serum, that suggest that severe COVID-19 is a result of cytokine release syndrome (CRS). This disorder is induced by cytokine storm [7]. Moreover, some studies analyzing cytokine profiles from COVID-19 patients indicated that cytokine storm is related directly with lung failure, multiorgan injury, and responsible for poor prognosis in severe COVID-19 course [16,17,18]. Assessment and management of COVID-19 depends on the course of the disease. The majority of the COVID-19 patients have mild or moderate disease symptoms. Patients with mild course usually recover at home, while moderate patients should be carefully monitored and occasionally hospitalized [19]. However, up to 5–10% of cases present severe disease course. Patients with severe pneumonia require hospitalization. Supportive care measures like ventilation oxygenation and fluid management remain the standard of care. However, for these patients, pharmacological treatment is definitely needed [20].

In adults, multisystem inflammatory syndrome (MIS-A) as a result of COVID-19 postinfectious processes observed 2–5 weeks later from probable COVID-19 infection is also disturbing. The syndrome is manifested by hyperinflammation and extrapulmonary organ dysfunction and can be fatal [21]. This will lead to the conclusion concerning not only the disease course but also the post COVID-19 recovery process. Even in patients with introduced recovery training program, the full recovery can last for months [22]. In this point, it should be outlined that investigational new drugs or more probable repurposing COVID-19 drugs is crucial not only in disease treatment but also in prevention of appearance of PIMS-TS or MIS-A. The drug repurposing is the crucial strategy, which lowers the costs, reduces the time to reach the market due to the fact that the phase I and II of clinical trials may not be required [23]. This article is aiming to analyze current pharmacological therapies for COVID-19 patients, focusing mainly on antiviral agents and drugs with immunomodulatory/anti-inflammatory properties, their mechanisms of action, and clinical trials developments.

## 2. Antiviral Agents

**Figure 2:**
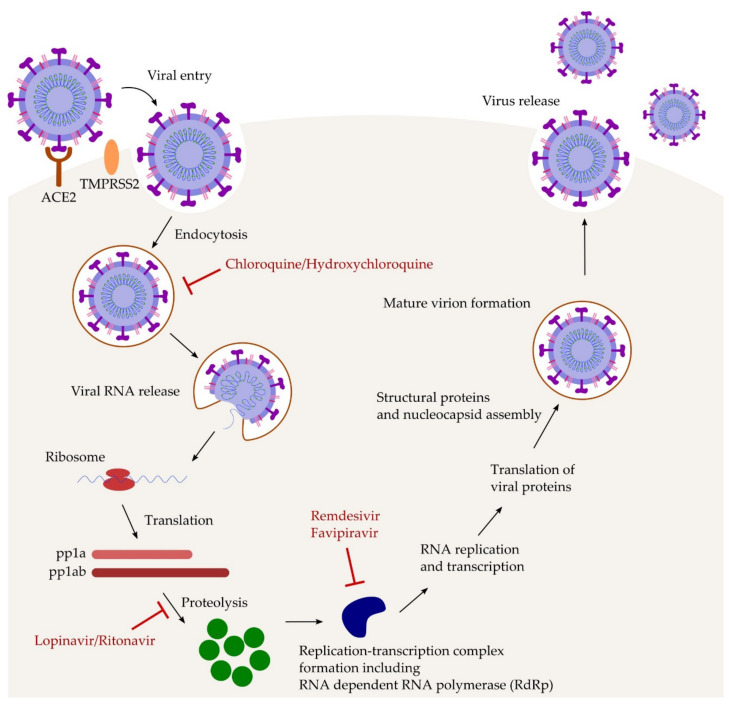
The mechanism of action of different antiviral agents against COVID-19. Chloroquine/Hydroxychloroquine have a wide range of action and can reduce the viral infectivity and immunomodulation or inhibit the viral entry or release into the host cell. Lopinavir inhibits protease that is essential for the production of mature virus particles. This drug is formulated in combination with ritonavir which increases the half-life of lopinavir. Remdesivir and favipiravir interfere with the RdRp that is crucial to the RNA replication and thus prevent the virus from multiplying. ACE2: angiotensin-converting enzyme 2; pp1a: replicase polyprotein pp1a; pp1ab: replicase polyprotein pp1ab; TMPRSS2: transmembrane serine protease 2.

Antiviral drugs seem to be an essential group of medicaments used in the COVID-19 treatment. The principal role of antiviral drugs is to block at any of the stages the viral replication cycle (Figure 2) [24]. Since the viral replication may be particularly active in the early COVID-19 stages, antiviral therapy administered shortly after symptoms onset may have the greatest impact on disease control process and lack of the progression to the hyperinflammatory state [25]. There are number of antiviral drugs available and approved for the treatment of different viral human infections. More than 50% of these drugs are used in immunodeficiency virus (HIV) infection, and the rest are used against influenza A and B, Ebola virus (EBOV), cytomegalovirus (CMV), hepatitis A (HAV) and C (HCV) and herpes simplex virus (HSV) [24]. Current available COVID-19 treatment options come from repurposing antiviral drugs using the advanced computational methods and assays for protein–protein interactions [26]. Performed clinical trials are focusing to evaluate the effectiveness of the available antiviral drugs against COVID-19 [27].

### 2.1. Remdesivir

**Figure 3:**
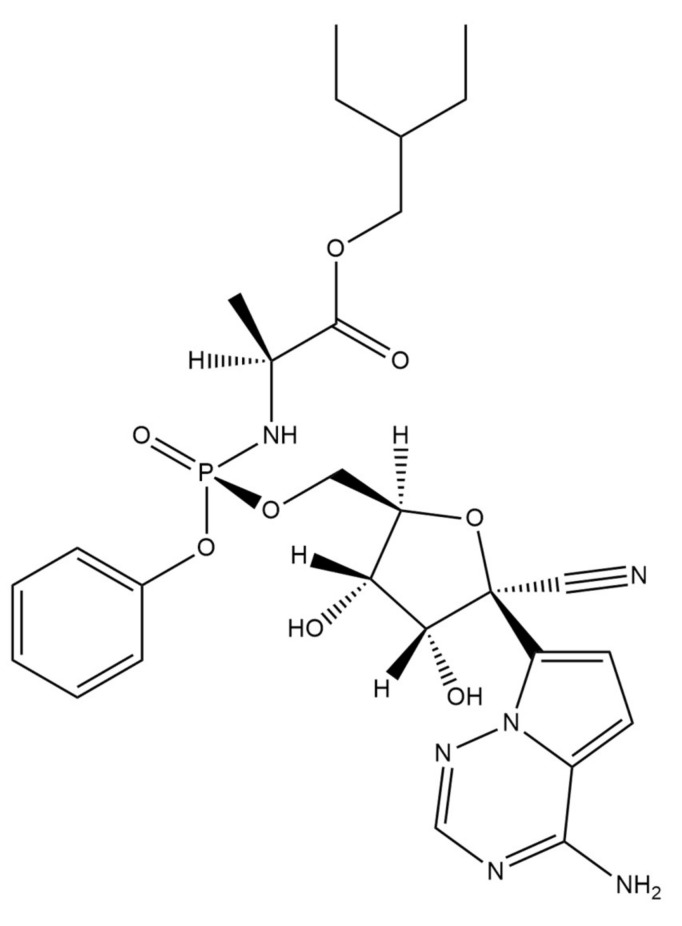
Chemical structure of remdesivir. The structure has been generated using InChI code available on https://pubchem.ncbi.nlm.nih.gov/ (accessed on 15 April 2021).

Remdesivir, formerly known as GS-5734, is a monophosphoramidate prodrug of an adenosine analog (Figure 3). It was initially developed and used in treatment of disease caused by EBOV and Marburg virus [28]. Remdesivir has been identified as a promising antiviral drug against a wide range of RNA viruses including Middle East respiratory syndrome coronavirus (MERS) and severe acute respiratory syndrome coronavirus 1 (SARS-CoV-1) [29,30,31]. What is more, in nonhuman primate studies, remdesivir initiated 12 h post MERS-CoV inoculation reduced virus replication in the lungs and severity of lung lesions [32].

Remdesivir, as a nucleotide analog, is able to inhibit RNA-dependent RNA polymerase (RdRp), essential for replication and transcription of viral RNA genome [33,34]. Remdesivir undergoes intracellular metabolic conversion to an active metabolite nucleoside triphosphate (NTP) analog, referred to as remdesivir triphosphate (RTP). Nucleoside analogs are synthetic compounds, which compete with endogenous nucleosides pools in integration into replicating viral RNA process. Furthermore, remdesivir monophosphate (RMP) mimics adenosine monophosphate (AMP) and forms typical Watson–Crick base pairs with uridine monophosphate (UMP) in the RNA template strand [35]. Although these synthetic compounds mimic their physiological equivalents, the inclusion of that analog molecule disturbs further molecular processes [36]. MERS-CoV studies have revealed that responsible for delayed chain termination upon incorporation into replicating RNA is remdesivir credible mechanism of action [30]. Gordon et al. have shown that in SARS-CoV-2, remdesivir causes the termination of RNA synthesis at three positions after the position where it is incorporated (*I* + 3). Premature termination of RNA synthesis abolishes subsequent transcription and translation processes that are essential to generate new virions. The mechanism was almost identical in SARS-CoV and MERS-CoV RdRps [37].

Since the beginning of the pandemic, many studies have been conducted using remdesivir and it has been identified as a potential COVID-19 therapeutic candidate due to its ability to inhibit SARS-CoV-2 in vitro [38]. The current recommendation is to administer in a 10-day regimen: 200 mg on day 1 followed by 100 mg daily for the following 9 days [39]. The first case report of SARS-CoV-2 infection confirmed in the United States and remdesivir application has been described by Holshue and colleagues [40]. The patient remained stable for the first 6 days after admission. However, the disease progressed with persistent fever and the need for oxygen supplementation was observed. Remdesivir was administered on day 7 due to patient’s clinical status worsening with significant clinical improvement over the next 24 h after administration. Beigel et al. [41] have conducted a double-blind, randomized, placebo-controlled trial of intravenous remdesivir in adults with COVID-19 lower respiratory tract infection symptoms. The data showed that the remdesivir was superior in comparison to placebo in reducing recovery time from 15 to 11 days. It suggested that remdesivir treatment could prevent progression to more severe respiratory disease by reducing the rate of serious adverse respiratory failure among remdesivir-treated patients’ events. Goldman et al. [42] in a randomized, open-label, without placebo trial among hospitalized patients with confirmed SARS-CoV-2 infection, oxygen saturation of 94% and radiologic evidence of pneumonia have demonstrated a clinical improvement. However, prolonged 10-days remdesivir therapy did not show a significant difference from 5-days regimen in severe but not requiring mechanical ventilation COVID-19 patients.

Many different clinical trials concerning application of remdesivir in COVID-19 treatment are undergoing and their characteristics are summarized in Table 1.

Remdesivir is the first drug that obtained U.S. Food and Drug Administration (FDA) approval for use in COVID-19 treatment. FDA approved the remdesivir for use in adult and pediatric patients above 12 years old and weighing at least 40 kg requiring hospitalization [48].

### 2.2. Favipiravir

**Figure 4:**
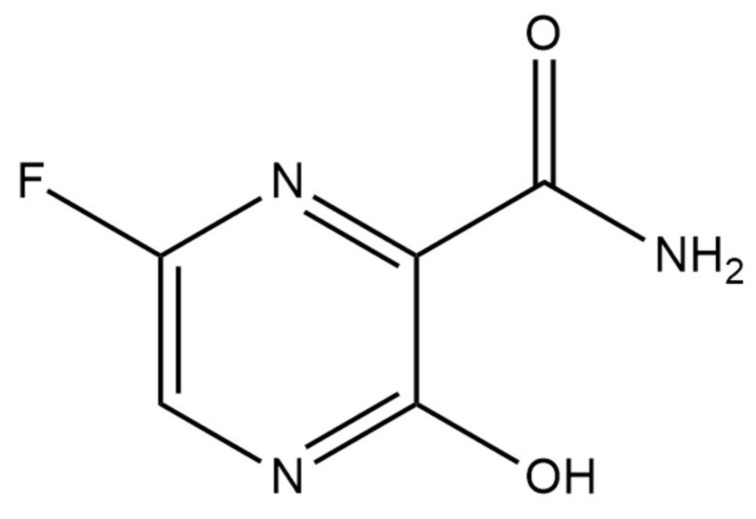
Chemical structure of favipiravir. The structure has been generated using InChI code available on https://pubchem.ncbi.nlm.nih.gov/ (accessed on 15 April 2021).

Favipiravir is a prodrug of purine nucleotide, which was discovered by chemical modification of pyrazine analog (Figure 4). It was initially used for screening anti-influenza virus activity. It was approved in 2014 in Japan, for the treatment of the new or reemerging pandemic influenza [49]. Although favipiravir was initially developed against influenza virus infections, it shows antiviral activities against other RNA viruses such as arenaviruses, filoviruses and bunyaviruses [50].

The drug is converted by intracellular phosphoribosylation into active favipiravir ribofuranosyl-5B-triphosphate (F-RTP). Then, it is recognized by RdRp as purine nucleotide and interferes with viral replication by getting incorporated into the viral RNA; thereby inhibits the activity of RdRp enzyme, which blocks the viral RNA synthesis [51]. Although the exact interaction mechanism of F-RTP with RdRp has not been explained yet, it is considered that F-RTP may be misincorporated in nascent viral RNA or binds to the RdRp catalytic domain. Thus, preventing further addition of nucleotides in the viral RNA replication process [52].

Several clinical trials have been launched in order to evaluate favipiravir efficacy and safety. In a multicenter, randomized controlled study patients with COVID-19 were randomly assigned into chloroquine and favipiravir groups. Patients who received favipiravir had lower duration of hospitalization than the chloroquine group. Furthermore, none of the patients in the favipiravir group needed mechanical ventilation [53]. In prospective, randomized, controlled, open-label, multicenter trial comparing favipiravir with umifenovir (an influenza virus entry inhibitor), favipiravir showed an improved clinical recovery rate, and reduced fever and cough duration compared to the umifenovir group in COVID-19 moderate symptoms patients. However, there was no statistical difference in supportive oxygen therapy or noninvasive mechanical ventilation [54].

At least 40 different clinical trials on the use of favipiravir in the treatment of COVID-19 are already registered in United States National Library of Medicine ClinicalTrials.gov [55]. The characteristics of main trials are summarized in Table 2.

### 2.3. Lopinavir/Ritonavir

**Figure 5:**
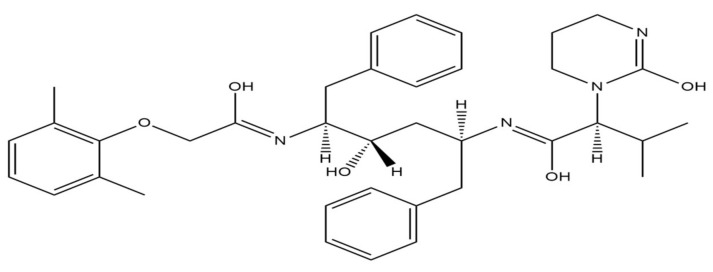
Chemical structure of lopinavir. The structure has been generated using InChI code available on https://pubchem.ncbi.nlm.nih.gov/ (accessed on 15 April 2021).

**Figure 6:**
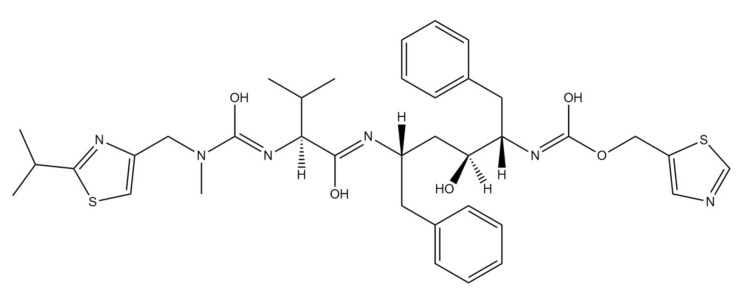
Chemical structure of ritonavir. The structure has been generated using InChI code available on https://pubchem.ncbi.nlm.nih.gov/ (accessed on 15 April 2021).

Lopinavir, an inhibitor of human immunodeficiency virus-1 (HIV-1) aspartate protease, has been used in HIV treatment (Figure 5) [61]. Lopinavir binds to the substrate site of the viral protease. Protease is responsible for the post-translational proteolysis of polyprotein precursor and the release of functional viral proteins. Thus, allowing them to function properly in replication/transcription and maturation processes. The consequence of inhibition is the production of immature virus particles [62]. Due to low lopinavir oral bioavailability and its extensive metabolism by the CYP3A4 isoenzyme, this drug is usually given in combination with low booster doses of ritonavir (Figure 6). That increases lopinavir plasma concentration by slowing its hepatic metabolism by inhibition of cytochrome P450 3A4 enzyme [63].

After the appearance of SARS in 2003, screening of approved drugs showed that the lopinavir has in vitro inhibitory effects against SARS-CoV [64]. Lopinavir also exhibits activity, both in vitro [65] and in an animal model [66], against MERS-CoV. Choy et al. also have shown that lopinavir can inhibit SARS-CoV-2 replication in Vero E6 cell line [67].

Further trials have been executed after promising in vitro studies. Cao et al. [68] have conducted a randomized, controlled, open-label trial involving hospitalized adult patients with confirmed SARS-CoV-2 infection. In this study, 199 COVID-19 patients with pneumonia symptoms with oxygen saturation ≤94% in ambient air received lopinavir/ritonavir or standard care. According to the study’s results, no differences between the lopinavir/ritonavir treatment and the standard care, in terms of clinical improvement, 28 days mortality rate, and detectable viral load were observed. The results from different trials have shown that lopinavir/ritonavir monotherapy is not an effective treatment for COVID-19 patients admitted to hospital. Furthermore, it was not associated with reductions of hospital stay duration [69]. The results of efficacy of lopinavir/ritonavir treatment in human coronavirus infections are not convincing, however, clinical trials are still ongoing and focusing on use of a combination of these drugs. The main ongoing clinical trials are listed in Table 3.

### 2.4. Chloroquine and Hydroxychloroquine

**Figure 7:**
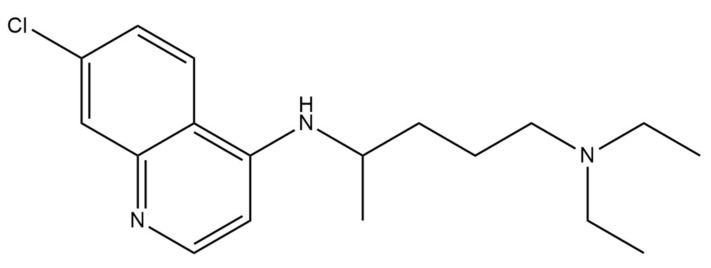
Chemical structure of chloroquine. The structure has been generated using InChI code available on https://pubchem.ncbi.nlm.nih.gov/ (accessed on 15 April 2021).

**Figure 8:**
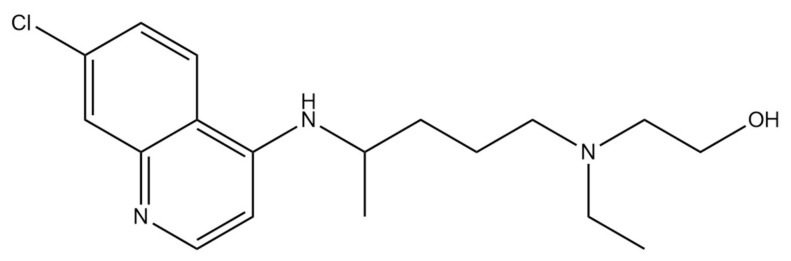
Chemical structure of hydroxychloroquine. The structure has been generated using InChI code available on https://pubchem.ncbi.nlm.nih.gov/ (accessed on 15 April 2021).

Chloroquine and hydroxychloroquine are authorized as antimalarial drugs and for the treatment of autoimmune diseases, including lupus and rheumatoid arthritis [75]. Their exact antiviral mechanisms of action are not fully understood, but evidence suggests that 4-aminoquinolines such as chloroquine (Figure 7) and hydroxychloroquine (Figure 8) have at least four mechanisms by which they can act. These mechanisms include inhibition of viral entry, inhibition of viral release into the host cell, reduction of viral infectivity and immunomodulation [76]. These drugs can be engulfed into endosomes and lysosomes, which lead to increased endosomal and lysosomal pH. It results in impaired release of the virus from the endosome or lysosome. As the virus release requires a low pH, the virus is unable to release its genetic material into the cell and replicate. Moreover, chloroquine and hydroxychloroquine decrease cytokine release, which could help mitigate the cytokine storm, which can occur in COVID-19 patients [77,78]. Chloroquine also promotes the cytosolic uptake of zinc, which has an antiviral effect by disrupting RdRp activity [79]. Chloroquine has been shown to interfere with terminal glycosylation of the cellular receptor ACE2. It negatively affects the virus–receptor binding process and abrogate the infection [80]. Fantini et al. [81] have proposed that chloroquine and hydroxychloroquine can prevent SARS-CoV-2 from binding with gangliosides, which in turn may inhibit virion contact with the ACE-2 receptor.

Another study has shown that hydroxychloroquine has been found to be more potent than chloroquine in in vitro inhibition of SARS-CoV-2 [82]. What is more, hydroxychloroquine clinical safety profile appears to be better than chloroquine (during long-term use) and allows to administer higher daily dose [83]. Gautret et al. [84] have conducted a small open-label nonrandomized trial performed on 36 COVID-19 patients. The obtained results showed the viral load was significantly lower in 20 cases treated with hydroxychloroquine at day 6 post inclusion compared to controls. Furthermore, azithromycin added to hydroxychloroquine was significantly more efficient in virus elimination process. According to Gao et al. [85] chloroquine phosphate is superior to the control treatment in inhibiting the COVID-19 exacerbation associated pneumonia. Moreover, it is improving lung imaging findings, promoting a virus negative conversion, and shortening the disease course. Furthermore, severe adverse reactions to chloroquine phosphate were not noted in trial patients. The different study was carried out on 84 COVID-19 patients receiving oral hydroxychloroquine and azithromycin. Patients were followed up with electrocardiography (ECG) and the results displayed the prolongation of QTc interval in comparison to baseline average. Eleven percent of patients experienced significant QTc prolongation, resulted in arrhythmia and sudden cardiac death. Therefore, hydroxychloroquine should be used with extreme precaution, particularly in patients taking QT-prolonging drugs and with existing comorbidities [86]. Moreover, FDA warns of use of hydroxychloroquine or chloroquine for COVID-19 outside of the hospital setting or a clinical trial due to risk of heart rhythm problems [87].

Despite this information, clinical trials concerning use of chloroquine and hydroxychloroquine in the treatment of COVID-19 are being conducted and some main examples are listed in Table 4.

## 3. Immunomodulatory/Anti-Inflammatory Agents

The later course of SARS-Cov-2 infection is likely to be related with excessive inflammatory and dysregulated immune responses with the exaggerate activation of inflammatory processes and the development of cytokine storm [93]. This is the basis for the immunomodulating/anti-inflammatory properties drugs’ implementation in the treatment of COVID-19 patients, as the immunomodulatory agents target the inflammatory response in patients’ lungs as well as the cytokine storm in severe cases [94]. A number of agents more commonly used in inflammatory conditions include both synthetic and biological medicaments, which are able to modulate specific inflammatory pathways by the IL-6 and IL-1 or janus kinase (JAK) and TNF-α inhibition is conducted [95]. However, despite the fact that preliminary results showed the immunomodulatory drug’s efficacy, the reports were diversified, and findings were not published yet [91].

### 3.1. Tocilizumab

Tocilizumab is a humanized monoclonal antibody against the interleukin-6 receptor (IL-6R). The drug is approved for the treatment of giant cell arteritis, rheumatoid arthritis, and CRS during chimeric antigen receptor T cell therapy (CAR-T) [96]. IL-6 is one of the major mediators of inflammatory and immune response initiated by infection. The increased IL-6 levels are found in more than half of COVID-19 patients. Data suggest that the IL-6 plays a pivotal role in guiding the inflammatory immune response at the level of pulmonary alveoli. This immune response causes the lung parenchyma injuries that significantly reduce respiratory function [97]. Increased IL-6 levels appear to be associated with respiratory failure resulting in the need for mechanical ventilation and escalating the mortality rate of COVID-19 patients [98].

IL-6R has two forms: membrane-bound interleukin-6 receptor (mIL-6R) and soluble interleukin-6 receptor (sIL-6R). IL-6 binds to sIL-6R to form a complex, which binds to glycoprotein 130 (gp130) on the cell membrane to complete signal transduction and plays a proinflammatory role [99]. Tocilizumab selectively and competitively binds sIL-6R and then inhibits the IL-6 mediated signal transduction. Thereby, drug reduces the availability of IL-6 and regulates immunological activity [100]. Therefore, some authors recommended the use of tocilizumab in critical COVID-19 cases with significantly elevated IL-6 [101].

To date, several independent trials have been initiated toward investigating the tocilizumab efficacy and safety. In one study, the drug usage has been retrospectively analyzed in 21 patients with severe and critical disease stage. Clinical data showed that the symptoms like hypoxygenmia and opacity visualized in computed tomography (CT) were improved immediately in most of the cases after the treatment with tocilizumab. The treatment was also associated with normalization of lymphocyte count and reduction of CRP levels, suggesting that tocilizumab could be an effective agent in COVID-19 treatment [99]. De Rossi et al. [100] have conducted a retrospective cohort study. Patients with COVID-19 related pneumonia at the early stage of respiratory failure were included. In this comparative study involving two groups of a total of 158 patients, 90 were treated with a single, low dose of tocilizumab and 68 patients were treated with the combination of hydroxychloroquine, lopinavir plus ritonavir. The study has found that the risk of death for patients treated with tocilizumab is 94% lower than the group with combination therapy. What is more, the drug effect on inflammatory indices is very quick, and low dose of tocilizumab is safe and not associated with adverse drug events. In conclusion, early treatment with tocilizumab may be helpful in preventing excessive inflammation and death in COVID-19 related to pneumonia. On the other hand, in some studies, tocilizumab has not shown a beneficial effect. Multicenter, randomized, open label, parallel group, superiority trial has been conducted in Brazil [96] among 129 adult patients with confirmed SARS-CoV-2. They experienced severe or critical COVID-19 form, with evidence of pulmonary infiltrates and were receiving supplemental oxygen or had been receiving mechanical ventilation for less than 24 h before analysis. They had also abnormal levels of at least two serum biomarkers (CRP, lactate dehydrogenase, D dimer or serum ferritin). The use of tocilizumab was not associated with an improvement in mechanical ventilation or decreased death rate in the tocilizumab group and in the control group. Adverse events were reported in 43% of patients, who received tocilizumab and 34% who did not receive tocilizumab. The drug is not superior to standard care alone in improving clinical outcomes and might increase mortality. Another study performed by Stone et al. [102] was a randomized, double-blind, placebo-controlled trial involving adult patients with confirmed SARS-CoV-2. Patients had pulmonary infiltrates, or a need for supplemental oxygen. The observed outcomes of completed clinical trials NCT04445272 and NCT04730323 were associated with intubation or death, clinical worsening, and discontinuation of supplemental oxygen among patients, who had been receiving drug at baseline. The data from completed studies do not allow to conclude that tocilizumab is an effective drug in moderately ill hospitalized patients.

Despite diverse information, many trials are currently underway, and details are presented in Table 5.

### 3.2. Anakinra

Anakinra is a high molecular weight recombinant of IL-1 receptor antagonist (IL-1RA), which is currently used in the treatment of rheumatoid arthritis and cryopyrin-associated periodic syndrome (CAPS). The drug has been found to be effective in severe sepsis in the patients’ subgroup with multiple organ dysfunction syndrome, in which the inflammasome pathway is also involved [108,109].

IL-1 is the prototypical inflammatory cytokine consisting of two distinct ligands (IL-1α and IL-1β), which bind the IL-1 type 1 receptor (IL-1R1) and numerous secondary inflammatory mediators such as cytokines, chemokines, and prostaglandins. IL-1α is present in epithelial and endothelial cells, while IL-1β is inducible in myeloid cells and released after cleavage by caspase-1. Anakinra as IL-1RA binds to the IL-1R and consequently prevents the binding of IL-1α as well as IL-1β to IL-1R1 and blocks their activity [110,111].

According to numerous studies, anakinra may be effective in treating severe forms of COVID-19. In the case report of critical COVID-19, anakinra was used after ineffective antiviral therapy. The invasive mechanical ventilation and hemodynamic support was required. After 72 h, rapid reduction of inflammatory markers and ferritin and increase of lymphocyte count were observed. Respiratory parameters improved by day 13 and at day 18 the patient was discharged from the intensive care unit (ICU) [112]. Kooistra et al. [113] have shown that anakinra can be effective in reducing clinical signs of hyperinflammation in critical forms of COVID-19. The conducted prospective cohort study involved mechanically ventilated COVID-19 patients with hyperinflammation. Among the group of patients enrolled in the study, 21 received anakinra and 39 standard care. Clinical outcomes were recorded until 28 days after alignment day. Anakinra treatment resulted in decreased clinical inflammatory response in comparison to control group, including a decrease in body temperature, ferritin, white blood cell counts, and procalcitonin plasma levels. Moreover, in the anakinra group, an improvement in kidney and liver function was observed. In another study, Huet and colleagues [114] confirmed the effectiveness of anakinra in the treatment of severe COVID-19 course. Fifty-two patients in the anakinra group were treated with anakinra and standard treatment, whereas the remaining 44 patients received standard treatments and supportive care. The results showed that in severe course of COVID-19 related to pneumonia, patients required oxygen therapy. A 10-day therapy with anakinra was related with significant reduction of both crucial factors: need of mechanical ventilation and mortality rate in comparison with the other group with similar features. The need for invasive mechanical ventilation or death occurred in 25% of patients in the anakinra group compared to 73% of patients observed in the comparison group. Moreover, the treatment with anakinra was not associated with serious side-effects [114]. In a different study, the authors examined whether anakinra could improve hospital patients’ outcomes with mild-to-moderate COVID-19 pneumonia. The performed multicenter, open-label randomized clinical trial included 116 patients: 59 were assigned to the anakinra group and 57 were in the standard care group. Results suggest that anakinra was not effective in reducing the need for mechanical or noninvasive ventilation or death rate in patients with COVID-19 and mild-to-moderate pneumonia. What is more, serious adverse events occurred in 46% patients in the anakinra group and 38% in the standard care group. Bacterial and fungal sepsis occurred in 11 patients in the anakinra group and in four patients in the standard care group [115].

Clinical trials concerning the usage of anakinra in COVID-19 are ongoing, but mainly focusing on severe courses, as shown in Table 6.

### 3.3. Baricitinib

**Figure 9:**
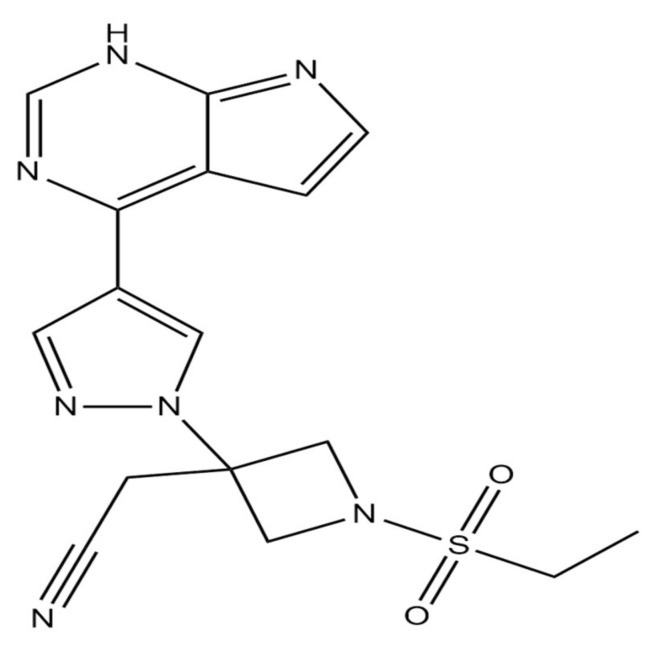
Chemical structure of baricitinib. The structure has been generated using InChI code available on https://pubchem.ncbi.nlm.nih.gov/ (accessed on 15 April 2021).

Baricitinib is a selective, efficient and safe janus kinase 1 (JAK1) and 2 (JAK2) inhibitor approved for usage in rheumatoid arthritis treatment (Figure 9) [121]. This enzyme transduces intracellular signals for cytokines and growth factors involved in processes such as hematopoiesis, inflammation and immune functions [122]. Baricitinib inhibits the intracellular signaling pathway of cytokines implicated in severe COVID-19, including IL-2, IL-6, IL-10, IFN-γ, and granulocyte-macrophage colony-stimulating factor (GM-CSF). Baricitinib also acts against SARS-CoV-2 by inhibition of both AP2-associated protein kinase 1 (AAK1) and cyclin G-associated kinase (GAK). Thus, preventing endocytosis and reducing viral assembly [123,124].

Studies on the usage of baricitinib in COVID-19 treatment are limited. The accessible ones concern a double-blinded, randomized, placebo-controlled trial evaluating baricitinib plus remdesivir in COVID-19 hospitalized adults. Patients, who received baricitinib and remdesivir recovered a day faster than patients who received remdesivir and placebo. The combination treatment also showed clinical benefits like accelerating improvement in clinical status among patients receiving noninvasive or high-flow oxygen ventilation. Their recovery time was shortened from 18 days to 10 days. Moreover, the combination therapy was associated with significantly lower incidence of adverse events [125]. The FDA has issued an emergency use authorization (EUA) for the baricitinib combined with remdesivir for the treatment of suspected or confirmed COVID-19 cases in hospitalized adults and pediatric patients aged two or older requiring supplemental oxygen, invasive mechanical ventilation, or extracorporeal membrane oxygenation (ECMO) [126].

Several trials concerning the use of baricitinib in COVID-19 treatment are currently underway and the examples are listed in Table 7.

### 3.4. Dexamethasone

**Figure 10:**
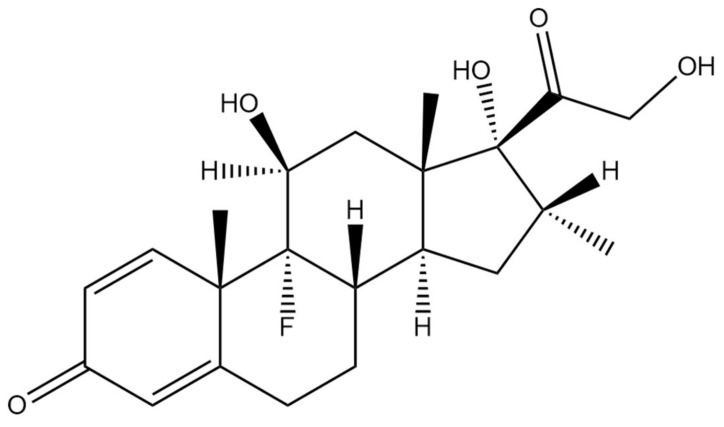
Chemical structure of dexamethasone. The structure has been generated using InChI code available on https://pubchem.ncbi.nlm.nih.gov/ (accessed on 15 April 2021).

Dexamethasone is a steroid compound, which belongs to the class of corticosteroids (Figure 10). This drug is widely used in the treatment of many inflammatory and autoimmune conditions, including asthma, chronic obstructive lung disease, rheumatic problems, different forms of allergies, some skin diseases and also in brain edema [132].

The mechanism of action of dexamethasone strictly depends on the dose used. At low doses, the drug binds to the glucocorticoid receptor (GCR) on the cell membrane, and the formation leads to translocation of the drug into the cell, where it moves to the nucleus. There, dexamethasone reversibly binds to several specific DNA sites resulting in transactivation and trans-repression of various gene transcription. Dexamethasone can inhibit the production of proinflammatory cytokines such as interleukin IL-1, IL-2, IL-6, IL-8, IFN-γ, vascular endothelial growth factor (VEGF) and prostaglandins. On the other hand, it can also induce the activation of anti-inflammatory cytokine synthesis, especially IL-10 and lipocortin-1 [133,134]. At high doses, the drug binds to membrane associated GCR cells such as T cells, resulting in impairment of receptor signaling and T cell mediated immune responses [133,135].

The number of studies on the drug usage in the COVID-19 treatment is very limited. However, it seems to be a promising drug. A controlled, open-label randomized evaluation of COVID-19 therapy (RECOVERY) trial concerning the use of dexamethasone in patients hospitalized with COVID-19 has been conducted. A total of 2104 patients were assigned to the dexamethasone group and 4321 to standard care. It has been shown that the use of dexamethasone for up to 10 days resulted in lower 28-day mortality rate among COVID-19 patients, who received either random invasive mechanical ventilation or oxygen alone. Among patients who were receiving oxygen, the use of dexamethasone was related with lowering the risk of invasive mechanical ventilation or, for patients receiving invasive mechanical ventilation, a greater chance of successful cessation. Additionally, patients in the dexamethasone group had a day shorter hospitalization in comparison to those in the standard care group and a greater probability of discharging within 28 days. However, there was no evidence that dexamethasone showed any beneficial effects in patients, who were not receiving respiratory support [136]. The CoDEX multicenter, randomized, open-label clinical trial, which included 299 patients with moderate or severe ARDS and COVID-19 has been conducted in 41 intensive care units (ICUs) in Brazil. Patients received dexamethasone plus standard care or standard care alone. The primary outcome was ventilator-free days during the first 28 days. Among patients with moderate or severe ARDS due to COVID-19, use of dexamethasone plus standard care vs. standard care alone resulted in significant increase in the number of ventilator-free days over 28 days. What is more, dexamethasone was not related to increased risk of adverse effects in this group of patients. Nevertheless, mortality rates were high and not significantly differ between groups, in contrast with the RECOVERY trial [137].

Dexamethasone is on the WHO guideline on COVID-19 drugs, however, it is suggested not to use corticosteroids in the treatment of patients with non-severe COVID-19 form [138]. Due to the limited data, further research is needed. Clinical trials are ongoing and some examples with short characteristics are listed in Table 8.

## 4. Conclusions and Future Perspectives

Currently, decision makers of countries around the world are taking precautions against spreading COVID-19. The scientific community is focusing on research aimed at repurposing of effective drugs against the SARS-CoV-2 virus. To date, many reports have been published on specific drugs’ efficacies and trials have been focusing on drugs with antiviral and immunomodulatory/anti-inflammatory effects. Moreover, several of these agents were repurposed to face the new COVID-19 health emergency. Overall, the results indicate that antiviral therapies have the greatest effect in the early stages of the disease, while immunomodulatory/anti-inflammatory agents are more effective in the later stages of the disease. A combination of these types of drugs also seems to be a good approach. However, the results of some studies are inconclusive or contradictory. Therefore, for the greater safety of COVID-19 patients, further clinical trials and large randomized controlled studies are required to verify their effective role, safety profile and side effects. In regard to appearance of PIMS-TS and MIS-A, the role of antiviral drugs remains unclear. PIMS-TS and MIS-A are clinically distinct from acute COVID-19, that may represent a post-COVID-19 immunological disease. Furthermore, there is no evidence concerning which treatment will be beneficial and prevent the appearance of PIMS-TS and MIS-A, so the recovery trials are needed. It should also not be forgotten that vaccines are an important aspect of combating the SARS-CoV-2 pandemic. Vaccines that are authorized and recommended are developed by BioNTech and Pfizer, Moderna, AstraZeneca and Johnson and Johnson. It is believed that the use of appropriate drugs depends on the severity of COVID-19 form and a global vaccination campaign will stop and overcome the pandemic that we have been facing for over a year now.

## Figures and Tables

**Table 1:** Characteristics of main clinical trials involving the use of remdesivir in COVID-19 patients.

ID	Official Title	Phase	Recruitment Status	Location	Reference
NCT04410354	A phase 2, randomized, double-blinded, placebo-controlled study on efficacy and safety of oral merimepodib in combination with intravenous remdesivir in adult patients with advanced coronavirus disease 2019 (COVID-19)	Phase 2	Terminated due to the failure to meet primary endpoint	United States	[43]
NCT04575064	An international randomized trial of additional COVID-19 treatments for hospitalized patients, who are receiving the local Standard Care—WHO-SOLIDARITY-GERMANY	Phase 2/3	Active, not recruiting	Germany	[44]
NCT04492475	A multicenter, adaptive, randomized blinded controlled trial on safety and efficacy of investigational COVID-19 therapeutics in hospitalized adults (ACTT-3)	Phase 3	Completed	United States	[45]
NCT04678739	Efficacy and safety of remdesivir and tociluzumab for the management of severe COVID-19 cases: a randomized controlled trial	Phase 3	Recruiting	Bangladesh	[46]
NCT04501952	A Phase 3 randomized, double-blinded placebo-controlled trial aiming to evaluate the efficacy and safety of remdesivir (GS-5734™) inCOVID-19. An outpatient setting	Phase 3	Recruiting	United States	[47]

**Table 2:** Characteristics of favipiravir clinical trials in patients with COVID-19.

ID	Official Title	Phase	Recruitment Status	Location	Reference
NCT04336904	A multicenter, randomized, double-blinded, placebo-controlled, phase III clinical study evaluating the efficacy and safety of favipiravir in the treatment of patients with COVID-19 moderate type	Phase 3	Active, not recruiting	Italy	[56]
NCT04558463	The effectivity and safety of favipiravir compared to oseltamivir as adjuvant therapy for COVID-19: an open label trial	Phase 3	Recruiting	Indonesia	[57]
NCT04349241	Efficacy and safety of Favipiravir in management of COVID-19	Phase 3	Completed	Egypt	[58]
NCT04499677	Favipiravir, lopinavir/ritonavir or combination therapy: a randomized, double blinded, 2 × 2 factorial placebo-controlled trial of early antiviral therapy in COVID-19	Phase 2	Recruiting	United Kingdom	[59]
NCT04600895	Favipiravir for patients with mild to moderate disease from novel coronavirus (COVID-19)	Phase 3	Recruiting	United States	[60]

**Table 3:** Lopinavir/Ritonavir trials in the treatment of COVID-19.

ID	Official Title	Phase	Recruitment Status	Location	Reference
NCT04499677	Favipiravir, lopinavir/ritonavir or combination therapy: a randomized, double blinded, 2x2 factorial placebo-controlled trial of early antiviral COVID-19 therapy	Phase 2	Recruiting	United Kingdom	[70]
NCT04372628	Trial of early therapies of COVID-19 non-hospitalized patients during outpatient window (TREAT NOW)	Phase 2	Recruiting	United States	[71]
NCT04403100	Hydroxychloroquine and lopinavir/ritonavir for hospitalization and mortality rate reduction in patients with COVID-19 mild disease symptoms: “The Hope Coalition”	Phase 3	Recruiting	Brazil	[72]
NCT04321174	COVID-19 ring-based prevention trial with lopinavir/ritonavir	Phase 3	Recruiting	Canada	[73]
NCT04738045	Comparative therapeutic efficacy and safety of remdesivir versus lopinavir/ritonavir and remdesivir combination in COVID-19 patients	Phase 4	Recruiting	Egypt	[74]

**Table 4:** Examples and clinical trials characteristics concerning the use of chloroquine and hydroxychloroquine in COVID-19 patients.

ID	Official Title	Phase	Recruitment Status	Location	Reference
NCT04420247	Multicentric pragmatic randomized controlled trial aiming to evaluate the efficacy of chloroquine or hydroxychloroquine for five days treatment of pneumonia caused by SARS-Cov-2—COVID-19	Phase 3	Completed	Brazil	[88]
NCT04342221	Randomized controlled trial of hydroxychloroquine versus placebo for the treatment of adult patients with acute coronavirus disease 2019—COVID-19	Phase 3	Recruiting	Germany	[89]
NCT04447534	Does zinc supplementation enhance the clinical efficacy of chloroquine/hydroxychloroquine in treatment of COVID-19?	Phase 3	Recruiting	Egypt	[90]
NCT04330144	A study of hydroxychloroquine as post exposure prophylaxis for SARS-CoV-2 (HOPE Trial)	Phase 3	Not yet recruiting	Not provided	[91]
NCT04344951	Chloroquine phosphate against infection by the novel coronavirus SARS-CoV-2 (COVID-19): the HOPE open-label, nonrandomized clinical trial (HOPE)	Phase 2	Recruiting	France	[92]

**Table 5:** The details of some clinical trials evaluating tocilizumab in COVID-19 treatment.

ID	Official Title	Phase	Recruitment Status	Location	Reference
NCT04445272	A multicenter, open-label clinical trial aiming to evaluate the effectiveness and safety of intravenous tocilizumab for treating patients with COVID-19 pneumonia: the BREATH-19 study	Phase 2	Completed	Spain	[103]
NCT04479358	COVIDOSE-2: a multicenter, randomized, controlled Phase 2 trial comparing early administration of low-dose tocilizumab to standard care in hospitalized patients with COVID-19 pneumonitis not requiring invasive ventilation	Phase 2	Recruiting	United States	[104]
NCT04412772	A randomized, controlled clinical trial on the safety and efficacy of tocilizumab for the treatment of severe COVID-19 cases	Phase 3	Recruiting	United States	[105]
NCT04730323	TOCILIZUMAB—an option for COVID-19 patients with associated cytokine release syndrome; A single center experience	Phase 4	Completed	Pakistan	[106]
NCT04332094	pilot, randomized, multicenter, open-label clinical trial of combined usage of hydroxychloroquine, azithromycin, and tocilizumab for the treatment of SARS-CoV-2 infection (COVID-19)	Phase 2	Recruiting	Spain	[107]

**Table 6:** Anakinra trials in COVID-19 treatment.

ID	Official Title	Phase	Recruitment Status	Location	Reference
NCT04339712	Efficiency in management of organ dysfunction associated with infection caused by the novel SARS-CoV-2 virus (COVID-19) through personalized immunotherapy approach: the ESCAPE clinical trial	Phase 2	Completed	Greece	[116]
NCT04148430	A Phase II study of IL-1 receptor antagonist—anakinra aiming to prevent severe neurotoxicity and cytokine release syndrome in patients receiving CD19-specific chimeric antigen receptor (CAR) T cells and to treat systemic inflammation associated with COVID-19	Phase 2	Recruiting	United States	[117]
NCT04680949	suPAR-guided anakinra treatment for validation of the risk and early management of severe respiratory failure caused by COVID-19: the SAVE-MORE double-blinded, randomized, Phase III confirmatory trial	Phase 3	Recruiting	Greece	[118]
NCT04443881	Clinical trial concerning the use of anakinra in cytokine storm syndrome secondary to COVID-19. A Phase 2/3, randomized, open-label, parallel group, 2-arm, multicenter study investigating the efficacy and safety of intravenous administrations of anakinra, an Interleukin-1(IL-1) receptor antagonist, added to standard care versus standard care in reducing hyper-inflammation and respiratory distress in patients with SARS-CoV-2	Phase 2/3	Recruiting	Spain	[119]
NCT04603742	Anakinra in adults with severe COVID-19 form and features of cytokine storm syndrome: a randomized, double-blinded, placebo-controlled trial	Phase 2	Not yet recruiting	United States	[120]

**Table 7:** Examples and characteristics of clinical trials concerning use of baricitinib in the COVID-19 therapy.

ID	Official Title	Phase	Recruitment Status	Location	Reference
NCT04421027	A randomized, double-blinded, placebo-controlled, parallel-group Phase 3 study of baricitinib in COVID-19 patients	Phase 3	Recruiting	United States	[127]
NCT04373044	A Phase II randomized double-blinded trial on baricitinib or placebo combined with antiviral therapy in patients with moderate and severe COVID-19 forms	Phase 2	Recruiting	United States	[128]
NCT04393051	BARICIVID-19 STUDY: multcenter, randomized, phase IIa clinical trial evaluating efficacy and tolerability of baricitinib as add-on treatment of COVID-19 in-patients in comparison to standard therapy	Phase 2	Not yet recruiting	Italy	[129]
NCT04321993	Treatment of moderate to severe coronavirus disease (COVID-19) cases among hospitalized patients	Phase 2	Recruiting	Canada	[130]
NCT04640168	A multicenter, adaptive, randomized blinded controlled trial on safety and efficacy of investigational therapeutics for the treatment of COVID-19 hospitalized adults (ACTT-4)	Phase 3	Recruiting	United States	[131]

**Table 8:** Examples of ongoing dexamethasone trials in the treatment of COVID-19.

ID	Official Title	Phase	Recruitment Status	Location	Reference
NCT04603729	Comparison of efficacy of dexamethasone and methylprednisolone in moderate to severe COVID-19 cases	Phase 3	Completed	Pakistan	[139]
NCT04663555	Effect of dexamethasone in patients with ARDS and COVID-19—prospective, multicenter, open-label, parallel-group, randomized controlled trial (REMED Trial)	Phase 4	Recruiting	Czech Republic	[140]
NCT04726098	Efficacy of low or high dose of dexamethasone in patients with respiratory failure caused by COVID-19	Phase 4	Recruiting	Spain	[141]
NCT04347980	dexamethasone combined with hydroxychloroquine compared to hydroxychloroquine alone for treatment of severe acute respiratory distress syndrome induced by coronavirus disease 19 (COVID-19): a multicenter, randomized controlled trial	Phase 3	Recruiting	France	[142]
NCT04452565	Randomized controlled phase 2/3 clinical trial of NA-831 alone or with atazanavir, or NA-831 with dexamethasone, or atazanavir with dexamethasone in the COVID-19 treatment	Phase 2/3	Recruiting	United States	[143]

## Abbreviations

AAK1	AP2-associated protein kinase 1
ACE2	Angiotensin-converting enzyme 2
AMP	Adenosine monophosphate
ARDS	Acute respiratory distress syndrome
CAPS	Cryopyrin-associated periodic syndrome
CAR-T	Chimeric antigen receptor T cell therapy
CD4	Cluster of differentiation 4
CD8	Cluster of differentiation 8
CMV	Cytomegalovirus
CRP	C-reactive protein
CRS	Cytokine release syndrome
CT	Computed tomography
CYP3A4	Human cytochrome P450 3A4
DIC	Disseminated intravascular coagulation
EBOV	Ebola virus
ECG	Electrocardiography
ECMO	Extracorporeal membrane oxygenation
EUA	Emergency use authorization
FDA	U.S. Food and Drug Administration
F-RTP	Favipiravir ribofuranosyl-5B-triphosphate
GAK	Cyclin G-associated kinase
GCR	Glucocorticoid receptor
G-CSF	Granulocyte colony-stimulating factor
GM-CSF	Granulocyte-macrophage colony-stimulating factor
gp130	Glycoprotein 130
HAV	hepatitis A
HCV	hepatitis C
HIV	Human immunodeficiency virus
HIV-1	Human immunodeficiency virus-1
HSV	Herpes simplex virus
ICU	Intensive care unit
IL-10	Interluekin-10
IL-1R1	IL-1 type 1 receptor
IL-1RA	IL-1 receptor antagonist
IL-2	Interluekin-2
IL-6	Interluekin-6
IL-6R	Interleukin-6 receptor
IL-7	Interluekin-7
IP-10	IFN-gamma-inducible protein 10
JAK	Janus kinase
JAK1	Janus kinase 1
JAK2	Janus kinase 2
MCP1	Monocyte chemoattractant protein 1
MERS	Middle East respiratory syndrome coronavirus
mIL-6R	Membrane-bound interleukin-6 receptor
MIP1α	Macrophage inflammatory protein 1α
MIS-A	Multisystem inflammatory syndrome
NK	Natural killer
NTP	Nucleoside triphosphate
PIMS-TS	Pediatric Inflammatory Multisystem Syndrome Temporally
pp1a	Replicase polyprotein pp1a
pp1ab	Replicase polyprotein pp1ab
RdRp RNA	dependent RNA polymerase
RECOVERY	Randomized evaluation of COVID-19 therapy
RMP	Remdesivir monophosphate
RTP	Remdesivir triphosphate
S	Spike
SARS-Cov-1	Severe acute respiratory syndrome coronavirus 1
SARS-CoV-2	Severe acute respiratory syndrome coronavirus 2
sIL-6R	Soluble interleukin-6 receptor
TMPRSS2	Transmembrane protease serine 2
TNF-α	Tumor necrosis factor-α
TSS	Toxic Shock Syndrome
UMP	Uridine monophosphate
VEGF	Vascular endothelial growth factor
WHO	World Health Organization

## References

[1] Du W., Han S., Li Q., Zhang Z. (2020). Epidemic update of COVID-19 in Hubei Province compared with other regions in China. Int. J. Infect. Dis. IJID Off. Publ. Int. Soc. Infect. Dis..

[2] World Health Organization WHO Coronavirus Disease (COVID-19) Dashboard. https://covid19.who.int/.

[3] Letko M., Marzi A., Munster V. (2020). Functional assessment of cell entry and receptor usage for SARS-CoV-2 and other lineage B betacoronaviruses. Nat. Microbiol..

[4] Huang Y., Yang C., Xu X.-F., Xu W., Liu S.-W. (2020). Structural and functional properties of SARS-CoV-2 spike protein: Potential antivirus drug development for COVID-19. Acta Pharmacol. Sin..

[5] Ma J., Qi X., Chen H., Li X., Zhang Z., Wang H., Sun L., Zhang L., Guo J., Morawska L. (2020). COVID-19 patients in earlier stages exhaled millions of SARS-CoV-2 per hour. Clin. Infect. Dis. Off. Publ. Infect. Dis. Soc. Am..

[6] Torres Acosta M.A., Singer B.D. (2020). Pathogenesis of COVID-19-induced ARDS: Implications for an ageing population. Eur. Respir. J..

[7] Huang C., Wang Y., Li X., Ren L., Zhao J., Hu Y., Zhang L., Fan G., Xu J., Gu X. (2020). Clinical features of patients infected with 2019 novel coronavirus in Wuhan, China. Lancet.

[8] Wu Y.-C., Chen C.-S., Chan Y.-J. (2020). The outbreak of COVID-19: An overview. J. Chin. Med. Assoc..

[9] Larsen J.R., Martin M.R., Martin J.D., Kuhn P., Hicks J.B. (2020). Modeling the Onset of Symptoms of COVID-19. Front. Public Health.

[10] Baj J., Karakuła-Juchnowicz H., Teresiński G., Buszewicz G., Ciesielka M., Sitarz E., Forma A., Karakuła K., Flieger W., Portincasa P. (2020). COVID-19: Specific and Non-Specific Clinical Manifestations and Symptoms: The Current State of Knowledge. J. Clin. Med..

[11] Abdelmaksoud A., Kroumpouzos G., Jafferany M., Lotti T., Sadoughifar R., Goldust M. (2020). COVID-19 in the pediatric population. Dermatol. Ther..

[12] She J., Liu L., Liu W. (2020). COVID-19 epidemic: Disease characteristics in children. J. Med. Virol..

[13] Harwood R., Allin B., Jones C.E., Whittaker E., Ramnarayan P., Ramanan A.V., Kaleem M., Tulloh R., Peters M.J., Almond S. (2021). A national consensus management pathway for paediatric inflammatory multisystem syndrome temporally associated with COVID-19 (PIMS-TS): Results of a national Delphi process. Lancet. Child Adolesc. Health.

[14] Chen N., Zhou M., Dong X., Qu J., Gong F., Han Y., Qiu Y., Wang J., Liu Y., Wei Y. (2020). Epidemiological and clinical characteristics of 99 cases of 2019 novel coronavirus pneumonia in Wuhan, China: A descriptive study. Lancet.

[15] Tan M., Liu Y., Zhou R., Deng X., Li F., Liang K., Shi Y. (2020). Immunopathological characteristics of coronavirus disease 2019 cases in Guangzhou, China. Immunology.

[16] Ruan Q., Yang K., Wang W., Jiang L., Song J. (2020). Clinical predictors of mortality due to COVID-19 based on an analysis of data of 150 patients from Wuhan, China. Intensive Care Med..

[17] Chen G., Wu D.I., Guo W., Cao Y., Huang D., Wang H., Wang T., Zhang X., Chen H., Yu H. (2020). Clinical and immunological features of severe and moderate coronavirus disease 2019. J. Clin. Investig..

[18] Gao Y., Li T., Han M., Li X., Wu D., Xu Y., Zhu Y., Liu Y., Wang X., Wang L. (2020). Diagnostic utility of clinical laboratory data determinations for patients with the severe COVID-19. J. Med. Virol..

[19] Gandhi R.T., Lynch J.B., del Rio C. (2020). Mild or Moderate Covid-19. N. Engl. J. Med..

[20] Gavriatopoulou M., Ntanasis-Stathopoulos I., Korompoki E., Fotiou D., Migkou M., Tzanninis I.-G., Psaltopoulou T., Kastritis E., Terpos E., Dimopoulos M.A. (2020). Emerging treatment strategies for COVID-19 infection. Clin. Exp. Med..

[21] Morris S.B., Schwartz N.G., Patel P., Abbo L., Beauchamps L., Balan S., Lee E.H., Paneth-Pollak R., Geevarughese A., Lash M.K. (2020). Case Series of Multisystem Inflammatory Syndrome in Adults Associated with SARS-CoV-2 Infection—United Kingdom and United States, March–August 2020. MMWR Morb. Mortal. Wkly. Rep..

[22] Huang C., Huang L., Wang Y., Li X., Ren L., Gu X., Kang L., Guo L., Liu M., Zhou X. (2021). 6-month consequences of COVID-19 in patients discharged from hospital: A cohort study. Lancet.

[23] Rosa S.G.V., Santos W.C. (2020). Clinical trials on drug repositioning for COVID-19 treatment. Rev. Panam. Salud Publica.

[24] Frediansyah A., Tiwari R., Sharun K., Dhama K., Harapan H. (2021). Antivirals for COVID-19: A critical review. Clin. Epidemiol. Glob. Health.

[25] Mitjà O., Clotet B. (2020). Use of antiviral drugs to reduce COVID-19 transmission. Lancet Glob. Health.

[26] Wang X., Guan Y. (2021). COVID-19 drug repurposing: A review of computational screening methods, clinical trials, and protein interaction assays. Med. Res. Rev..

[27] Richman D.D. (2020). Antiviral Drug Discovery to Address the COVID-19 Pandemic. mBio.

[28] Hendaus M.A. (2020). Remdesivir in the treatment of coronavirus disease 2019 (COVID-19): A simplified summary. J. Biomol. Struct. Dyn..

[29] Sheahan T.P., Sims A.C., Leist S.R., Schäfer A., Won J., Brown A.J., Montgomery S.A., Hogg A., Babusis D., Clarke M.O. (2020). Comparative therapeutic efficacy of remdesivir and combination lopinavir, ritonavir, and interferon beta against MERS-CoV. Nat. Commun..

[30] Agostini M.L., Andres E.L., Sims A.C., Graham R.L., Sheahan T.P., Lu X., Smith E.C., Case J.B., Feng J.Y., Jordan R. (2018). Coronavirus susceptibility to the antiviral remdesivir (GS-5734) is mediated by the viral polymerase and the proofreading exoribonuclease. mBio.

[31] Brown A.J., Won J.J., Graham R.L., Dinnon Iii K.H., Sims A.C., Feng J.Y., Cihlar T., Denison M.R., Baric R.S., Sheahan T.P. (2019). Broad spectrum antiviral remdesivir inhibits human endemic and zoonotic deltacoronaviruses with a highly divergent RNA dependent RNA polymerase. Antivir. Res..

[32] de Wit E., Feldmann F., Cronin J., Jordan R., Okumura A., Thomas T., Scott D., Cihlar T., Feldmann H. (2020). Prophylactic and therapeutic remdesivir (GS-5734) treatment in the rhesus macaque model of MERS-CoV infection. Proc. Natl. Acad. Sci. USA.

[33] Posthuma C.C., Te Velthuis A.J.W., Snijder E.J. (2017). Nidovirus RNA polymerases: Complex enzymes handling exceptional RNA genomes. Virus Res..

[34] Jiang Y., Yin W., Xu H.E. (2021). RNA-dependent RNA polymerase: Structure, mechanism, and drug discovery for COVID-19. Biochem. Biophys. Res. Commun..

[35] Kokic G., Hillen H.S., Tegunov D., Dienemann C., Seitz F., Schmitzova J., Farnung L., Siewert A., Höbartner C., Cramer P. (2021). Mechanism of SARS-CoV-2 polymerase stalling by remdesivir. Nat. Commun..

[36] Tchesnokov E.P., Feng J.Y., Porter D.P., Götte M. (2019). Mechanism of inhibition of Ebola virus RNA-dependent RNA polymerase by remdesivir. Viruses.

[37] Gordon C.J., Tchesnokov E.P., Woolner E., Perry J.K., Feng J.Y., Porter D.P., Götte M. (2020). Remdesivir is a direct-acting antiviral that inhibits RNA-dependent RNA polymerase from severe acute respiratory syndrome coronavirus 2 with high potency. J. Biol. Chem..

[38] Wang M., Cao R., Zhang L., Yang X., Liu J., Xu M., Shi Z., Hu Z., Zhong W., Xiao G. (2020). Remdesivir and chloroquine effectively inhibit the recently emerged novel coronavirus (2019-nCoV) in vitro. Cell Res..

[39] Wu R., Wang L., Kuo H.-C.D., Shannar A., Peter R., Chou P.J., Li S., Hudlikar R., Liu X., Liu Z. (2020). An Update on Current Therapeutic Drugs Treating COVID-19. Curr. Pharmacol. Rep..

[40] Holshue M.L., DeBolt C., Lindquist S., Lofy K.H., Wiesman J., Bruce H., Spitters C., Ericson K., Wilkerson S., Tural A. (2020). First case of 2019 novel coronavirus in the United States. N. Engl. J. Med..

[41] Beigel J.H., Tomashek K.M., Dodd L.E., Mehta A.K., Zingman B.S., Kalil A.C., Hohmann E., Chu H.Y., Luetkemeyer A., Kline S. (2020). Remdesivir for the Treatment of Covid-19—Final Report. N. Engl. J. Med..

[42] Goldman J.D., Lye D.C.B., Hui D.S., Marks K.M., Bruno R., Montejano R., Spinner C.D., Galli M., Ahn M.-Y., Nahass R.G. (2020). Remdesivir for 5 or 10 Days in Patients with Severe Covid-19. N. Engl. J. Med..

[43] ClinicalTrials.gov A Phase 2, Randomized, Double-Blind, Placebo-Controlled Study of the Efficacy and Safety of Oral Merimepodib in Combination with Intravenous Remdesivir in Adult Patients With Advanced Coronavirus Disease 2019 (COVID-19). NCT04410354.

[44] ClinicalTrials.gov An International Randomized Trial of Additional Treatments for COVID-19 in Hospitalized Patients Who Are All Receiving the Local Standard of Care—WHO-SOLIDARITY-GERMANY. NCT04575064.

[45] ClinicalTrials.gov A Multicenter, Adaptive, Randomized Blinded Controlled Trial of the Safety and Efficacy of Investigational Therapeutics for the Treatment of COVID-19 in Hospitalized Adults (ACTT-3). NCT04492475.

[46] ClinicalTrials.gov Efficacy and Safety of Remdesivir and Tociluzumab for the Management of Severe COVID-19: A Randomized Controlled Trial. NCT04678739.

[47] ClinicalTrials.gov A Phase 3 Randomized, Double-Blind Placebo-Controlled Trial to Evaluate the Efficacy and Safety of Remdesivir (GS-5734™) Treatment of COVID-19 in an Outpatient Setting. NCT04501952.

[48] The U.S. Food and Drug Administration FDA Approves First Treatment for COVID-19. https://www.fda.gov/news-events/press-announcements/fda-approves-first-treatment-covid-19.

[49] Shiraki K., Daikoku T. (2020). Favipiravir, an anti-influenza drug against life-threatening RNA virus infections. Pharmacol. Ther..

[50] Furuta Y., Komeno T., Nakamura T. (2017). Favipiravir (T-705), a broad spectrum inhibitor of viral RNA polymerase. Proc. Jpn. Acad. Ser. B Phys. Biol. Sci..

[51] Sanders J.M., Monogue M.L., Jodlowski T.Z., Cutrell J.B. (2020). Pharmacologic treatments for coronavirus disease 2019 (COVID-19): A review. JAMA.

[52] Zhu W., Chen C.Z., Gorshkov K., Xu M., Lo D.C., Zheng W. (2020). RNA-Dependent RNA Polymerase as a Target for COVID-19 Drug Discovery. SLAS Discov. Adv. Sci. Drug Discov..

[53] Dabbous H.M., Abd-Elsalam S., El-Sayed M.H., Sherief A.F., Ebeid F.F.S., El Ghafar M.S.A., Soliman S., Elbahnasawy M., Badawi R., Tageldin M.A. (2021). Efficacy of favipiravir in COVID-19 treatment: A multi-center randomized study. Arch. Virol..

[54] Chen C., Huang J., Cheng Z., Wu J., Chen S., Zhang Y., Chen B., Lu M., Luo Y., Zhang J. (2020). Favipiravir versus arbidol for COVID-19: A randomized clinical trial. medRxiv.

[55] ClinicalTrials.gov The U.S. National Library of Medicine. Fa Vipiravir. https://clinicaltrials.gov/ct2/results?recrs=&cond=Covid19&term=Favipiravir&cntry=&state=&city=&dist=.

[56] ClinicalTrials.gov A Multi-Center, Randomized, Double-Blind, Placebo-Controlled, Phase III Clinical Study Evaluating the Efficacy and Safety of Favipiravir in the Treatment of Patients With COVID-19-Moderate Type. NCT04336904.

[57] ClinicalTrials.gov The Effectivity and Safety of Favipiravir Compared to Oseltamivir as Adjuvant Therapy for COVID-19: An Open Label Trial. NCT04558463.

[58] ClinicalTrials.gov Efficacy and Safety of Favipiravir in Management of COVID-19. NCT04349241.

[59] ClinicalTrials.gov Favipiravir, Lopinavir/Ritonavir or Combination Therapy: A Randomised, Double Blind, 2x2 Factorial Placebo-Controlled Trial of Early Antiviral Therapy in COVID-19. NCT04499677.

[60] ClinicalTrials.gov Favipiravir for Patients with Mild to Moderate Disease from Novel Coronavirus (COVID-19). NCT04600895.

[61] Chandwani A., Shuter J. (2008). Lopinavir/ritonavir in the treatment of HIV-1 infection: A review. Ther. Clin. Risk Manag..

[62] Bolcato G., Bissaro M., Pavan M., Sturlese M., Moro S. (2020). Targeting the coronavirus SARS-CoV-2: Computational insights into the mechanism of action of the protease inhibitors lopinavir, ritonavir and nelfinavir. Sci. Rep..

[63] Chu C.M., Cheng V.C.C., Hung I.F.N., Wong M.M.L., Chan K.H., Chan K.S., Kao R.Y.T., Poon L.L.M., Wong C.L.P., Guan Y. (2004). Role of lopinavir/ritonavir in the treatment of SARS: Initial virological and clinical findings. Thorax.

[64] Wu C.-Y., Jan J.-T., Ma S.-H., Kuo C.-J., Juan H.-F., Cheng Y.-S.E., Hsu H.-H., Huang H.-C., Wu D., Brik A. (2004). Small molecules targeting severe acute respiratory syndrome human coronavirus. Proc. Natl. Acad. Sci. USA.

[65] De Wilde A.H., Jochmans D., Posthuma C.C., Zevenhoven-Dobbe J.C., Van Nieuwkoop S., Bestebroer T.M., Van Den Hoogen B.G., Neyts J., Snijder E.J. (2014). Screening of an FDA-approved compound library identifies four small-molecule inhibitors of Middle East respiratory syndrome coronavirus replication in cell culture. Antimicrob. Agents Chemother..

[66] Chan J.F.-W., Yao Y., Yeung M.-L., Deng W., Bao L., Jia L., Li F., Xiao C., Gao H., Yu P. (2015). Treatment with lopinavir/ritonavir or interferon-β1b improves outcome of MERS-CoV infection in a nonhuman primate model of common marmoset. J. Infect. Dis..

[67] Choy K.-T., Wong A.Y.-L., Kaewpreedee P., Sia S.F., Chen D., Hui K.P.Y., Chu D.K.W., Chan M.C.W., Cheung P.P.-H., Huang X. (2020). Remdesivir, lopinavir, emetine, and homoharringtonine inhibit SARS-CoV-2 replication in vitro. Antivir. Res..

[68] Cao B., Wang Y., Wen D., Liu W., Wang J., Fan G., Ruan L., Song B., Cai Y., Wei M. (2020). A Trial of Lopinavir-Ritonavir in Adults Hospitalized with Severe Covid-19. N. Engl. J. Med..

[69] Horby P.W., Mafham M., Bell J.L., Linsell L., Staplin N., Emberson J., Palfreeman A., Raw J., Elmahi E., Prudon B. (2020). Lopinavir-ritonavir in patients admitted to hospital with COVID-19 (RECOVERY): A randomised, controlled, open-label, platform trial. Lancet.

[70] ClinicalTrials.gov FLARE: Favipiravir +/- Lopinavir: A RCT of Early Antivirals. NCT04499677.

[71] ClinicalTrials.gov Trial of Early Therapies during Non-hospitalized Outpatient Window (TREAT NOW) for COVID-19. NCT04372628.

[72] ClinicalTrials.gov Hydroxychloroquine and Lopinavir/Ritonavir for Hospitalization and Mortality Reduction in Patients with COVID-19 and Mild Disease Symptoms: “The Hope Coalition”. NCT04403100.

[73] ClinicalTrials.gov COVID-19 Ring-based Prevention Trial with Lopinavir/Ritonavir. NCT04321174.

[74] ClinicalTrials.gov Comparative Therapeutic Efficacy and Safety of Remdesivir Versus Lopinavir/Ritonavir and Remdesivir Combination in COVID-19 Patients. NCT04738045.

[75] Khuroo M.S. (2020). Chloroquine and hydroxychloroquine in coronavirus disease 2019 (COVID-19). Facts, fiction and the hype: A critical appraisal. Int. J. Antimicrob. Agents.

[76] Tripathy S., Dassarma B., Roy S., Chabalala H., Matsabisa M.G. (2020). A review on possible modes of action of chloroquine/hydroxychloroquine: Repurposing against SAR-CoV-2 (COVID-19) pandemic. Int. J. Antimicrob. Agents.

[77] Huang Z., Srinivasan S., Zhang J., Chen K., Li Y., Li W., Quiocho F.A., Pan X. (2012). Discovering thiamine transporters as targets of chloroquine using a novel functional genomics strategy. PLoS Genet..

[78] Pastick K.A., Okafor E.C., Wang F., Lofgren S.M., Skipper C.P., Nicol M.R., Pullen M.F., Rajasingham R., McDonald E.G., Lee T.C. (2020). Review: Hydroxychloroquine and Chloroquine for Treatment of SARS-CoV-2 (COVID-19). Open Forum Infect. Dis..

[79] Skalny A.V., Rink L., Ajsuvakova O.P., Aschner M., Gritsenko V.A., Alekseenko S.I., Svistunov A.A., Petrakis D., Spandidos D.A., Aaseth J. (2020). Zinc and respiratory tract infections: Perspectives for COVID-19 (Review). Int. J. Mol. Med..

[80] Vincent M.J., Bergeron E., Benjannet S., Erickson B.R., Rollin P.E., Ksiazek T.G., Seidah N.G., Nichol S.T. (2005). Chloroquine is a potent inhibitor of SARS coronavirus infection and spread. Virol. J..

[81] Fantini J., Di Scala C., Chahinian H., Yahi N. (2020). Structural and molecular modelling studies reveal a new mechanism of action of chloroquine and hydroxychloroquine against SARS-CoV-2 infection. Int. J. Antimicrob. Agents.

[82] Yao X., Ye F., Zhang M., Cui C., Huang B., Niu P., Liu X., Zhao L., Dong E., Song C. (2020). In Vitro Antiviral Activity and Projection of Optimized Dosing Design of Hydroxychloroquine for the Treatment of Severe Acute Respiratory Syndrome Coronavirus 2 (SARS-CoV-2). Clin. Infect. Dis. Off. Publ. Infect. Dis. Soc. Am..

[83] Marmor M.F., Kellner U., Lai T.Y.Y., Melles R.B., Mieler W.F. (2016). Recommendations on Screening for Chloroquine and Hydroxychloroquine Retinopathy (2016 Revision). Ophthalmology.

[84] Gautret P., Lagier J.-C., Parola P., Hoang V.T., Meddeb L., Mailhe M., Doudier B., Courjon J., Giordanengo V., Vieira V.E. (2020). Hydroxychloroquine and azithromycin as a treatment of COVID-19: Results of an open-label non-randomized clinical trial. Int. J. Antimicrob. Agents.

[85] Gao J., Tian Z., Yang X. (2020). Breakthrough: Chloroquine phosphate has shown apparent efficacy in treatment of COVID-19 associated pneumonia in clinical studies. Biosci. Trends.

[86] Chorin E., Dai M., Shulman E., Wadhwani L., Bar-Cohen R., Barbhaiya C., Aizer A., Holmes D., Bernstein S., Spinelli M. (2020). The QT interval in patients with COVID-19 treated with hydroxychloroquine and azithromycin. Nat. Med..

[87] The U.S. Food and Drug Administration FDA Cautions against Use of Hydroxychloroquine or Chloroquine for COVID-19 Outside of the Hospital Setting or a Clinical Trial Due to Risk of Heart Rhythm Problems. https://www.fda.gov/drugs/drug-safety-and-availability/fda-cautions-against-use-hydroxychloroquine-or-chloroquine-covid-19-outside-hospital-setting-or.

[88] ClinicalTrials.gov Multicentric Pragmatic Randomized Controled Trial to Evaluate the Efficacy Chloroquine or Hydroxychloroquine for Five Days in Treating Pneumonia Caused by SARS-Cov-2—COVID-19. NCT04420247.

[89] ClinicalTrials.gov Randomized Controlled Trial of Hydroxychloroquine Versus Placebo for the Treatment of Adult Patients with Acute Coronavirus Disease 2019—COVID-19. NCT04342221.

[90] ClinicalTrials.gov Does Zinc Supplementation Enhance the Clinical Efficacy of Chloroquine/Hydroxychloroquine in Treatment of COVID-19?. NCT04447534.

[91] ClinicalTrials.gov A Study of Hydroxychloroquine as Post Exposure Prophylaxis for SARS-CoV-2 (HOPE Trial). NCT04330144.

[92] ClinicalTrials.gov Chloroquine Phosphate against Infection by the Novel Coronavirus SARS-CoV-2 (COVID-19): The HOPE Open-Label, Non Randomized Clinical Trial (HOPE). NCT04344951.

[93] Song P., Li W., Xie J., Hou Y., You C. (2020). Cytokine storm induced by SARS-CoV-2. Clin. Chim. Acta Int. J. Clin. Chem..

[94] Burrage D.R., Koushesh S., Sofat N. (2020). Immunomodulatory Drugs in the Management of SARS-CoV-2. Front. Immunol..

[95] Razmi M., Hashemi F., Gheytanchi E., Dehghan Manshadi M., Ghods R., Madjd Z. (2020). Immunomodulatory-based therapy as a potential promising treatment strategy against severe COVID-19 patients: A systematic review. Int. Immunopharmacol..

[96] Veiga V.C., Prats J.A.G.G., Farias D.L.C., Rosa R.G., Dourado L.K., Zampieri F.G., Machado F.R., Lopes R.D., Berwanger O., Azevedo L.C.P. (2021). Effect of tocilizumab on clinical outcomes at 15 days in patients with severe or critical coronavirus disease 2019: Randomised controlled trial. BMJ.

[97] Grifoni E., Valoriani A., Cei F., Lamanna R., Gelli A.M.G., Ciambotti B., Vannucchi V., Moroni F., Pelagatti L., Tarquini R. (2020). Interleukin-6 as prognosticator in patients with COVID-19. J. Infect..

[98] Zhang Z.-L., Hou Y.-L., Li D.-T., Li F.-Z. (2020). Laboratory findings of COVID-19: A systematic review and meta-analysis. Scand. J. Clin. Lab. Investig..

[99] Xu X., Han M., Li T., Sun W., Wang D., Fu B., Zhou Y., Zheng X., Yang Y., Li X. (2020). Effective treatment of severe COVID-19 patients with tocilizumab. Proc. Natl. Acad. Sci. USA.

[100] De Rossi N., Scarpazza C., Filippini C., Cordioli C., Rasia S., Mancinelli C.R., Rizzoni D., Romanelli G., Cossi S., Vettoretto N. (2020). Early use of low dose tocilizumab in patients with COVID-19: A retrospective cohort study with a complete follow-up. EClinicalMedicine.

[101] Zhang C., Wu Z., Li J.-W., Zhao H., Wang G.-Q. (2020). Cytokine release syndrome in severe COVID-19: Interleukin-6 receptor antagonist tocilizumab may be the key to reduce mortality. Int. J. Antimicrob. Agents.

[102] Stone J.H., Frigault M.J., Serling-Boyd N.J., Fernandes A.D., Harvey L., Foulkes A.S., Horick N.K., Healy B.C., Shah R., Bensaci A.M. (2020). Efficacy of Tocilizumab in Patients Hospitalized with Covid-19. N. Engl. J. Med..

[103] ClinicalTrials.gov A Multicentre, Open-Label Clinical Trial to Evaluate the Effectiveness and Safety of Intravenous Tocilizumab for Treating Patients with COVID-19 Pneumonia: The BREATH-19 Study. NCT04445272.

[104] ClinicalTrials.gov COVIDOSE-2: A Multi-Center, Randomized, Controlled Phase 2 Trial Comparing Early Administration of Low-Dose Tocilizumab to Standard of Care in Hospitalized Patients with COVID-19 Pneumonitis Not Requiring Invasive Ventilation. NCT04479358.

[105] ClinicalTrials.gov A Randomized, Controlled Clinical Trial of the Safety and Efficacy of Tocilizumab for the Treatment of Severe COVID-19. NCT04412772.

[106] ClinicalTrials.gov TOCILIZUMAB—An Option for Patients with COVID-19 Associated Cytokine Release Syndrome; A Single Center Experience. NCT04730323.

[107] ClinicalTrials.gov Pilot, Randomized, Multicenter, Open-Label Clinical Trial of Combined Use of Hydroxychloroquine, Azithromycin, and Tocilizumab for the Treatment of SARS-CoV-2 Infection (COVID-19). NCT04332094.

[108] Shakoory B., Carcillo J.A., Chatham W.W., Amdur R.L., Zhao H., Dinarello C.A., Cron R.Q., Opal S.M. (2016). Interleukin-1 Receptor Blockade Is Associated with Reduced Mortality in Sepsis Patients with Features of Macrophage Activation Syndrome: Reanalysis of a Prior Phase III Trial. Crit. Care Med..

[109] Rajasekaran S., Kruse K., Kovey K., Davis A.T., Hassan N.E., Ndika A.N., Zuiderveen S., Birmingham J. (2014). Therapeutic role of anakinra, an interleukin-1 receptor antagonist, in the management of secondary hemophagocytic lymphohistiocytosis/sepsis/multiple organ dysfunction/macrophage activating syndrome in critically ill children. Pediatr. Crit. Care Med..

[110] Cavalli G., Dinarello C.A. (2018). Anakinra Therapy for Non-cancer Inflammatory Diseases. Front. Pharmacol..

[111] King A., Vail A., O’Leary C., Hannan C., Brough D., Patel H., Galea J., Ogungbenro K., Wright M., Pathmanaban O. (2020). Anakinra in COVID-19: Important considerations for clinical trials. Lancet Rheumatol..

[112] Filocamo G., Mangioni D., Tagliabue P., Aliberti S., Costantino G., Minoia F., Bandera A. (2020). Use of anakinra in severe COVID-19: A case report. Int. J. Infect. Dis..

[113] Kooistra E.J., Waalders N.J.B., Grondman I., Janssen N.A.F., de Nooijer A.H., Netea M.G., van de Veerdonk F.L., Ewalds E., van der Hoeven J.G., Kox M. (2020). Anakinra treatment in critically ill COVID-19 patients: A prospective cohort study. Crit. Care.

[114] Huet T., Beaussier H., Voisin O., Jouveshomme S., Dauriat G., Lazareth I., Sacco E., Naccache J.-M., Bézie Y., Laplanche S. (2020). Anakinra for severe forms of COVID-19: A cohort study. Lancet Rheumatol..

[115] Tharaux P.-L., Pialoux G., Pavot A., Mariette X., Hermine O., Resche-Rigon M., Porcher R., Ravaud P., Bureau S., Dougados M. (2021). Effect of anakinra versus usual care in adults in hospital with COVID-19 and mild-to-moderate pneumonia (CORIMUNO-ANA-1): A randomised controlled trial. Lancet Respir. Med..

[116] ClinicalTrials.gov Efficiency in Management of Organ Dysfunction Associated with Infection by the Novel SARS-CoV-2 Virus (COVID-19) Through a Personalized Immunotherapy Approach: The ESCAPE Clinical Trial. NCT04339712.

[117] ClinicalTrials.gov A Phase II Study of IL-1 Receptor Antagonist Anakinra to Prevent Severe Neurotoxicity and Cytokine Release Syndrome in Patients Receiving CD19-Specific Chimeric Antigen Receptor (CAR) T Cells and to Treat Systemic Inflammation Associated with COVID-19. NCT04148430.

[118] ClinicalTrials.gov suPAR-Guided Anakinra Treatment for Validation of the Risk and Early Management of Severe Respiratory Failure by COVID-19: The SAVE-MORE Double-Blind, Randomized, Phase III Confirmatory Trial. NCT04680949.

[119] ClinicalTrials.gov Clinical Trial of the Use of Anakinra in Cytokine Storm Syndrome Secondary to Covid-19. A Phase 2/3, Randomized, Open-Label, Parallel Group, 2-arm, Multicenter Study Investigating the Efficacy and Safety of Intravenous Administrations of Anakinra, an Interleukin-1(IL-1) Receptor Antagonist, Added to Standard of Care, Versus Standard of Care, in Reducing Hyper-inflammation and Respiratory Distress in Patients With SARS-CoV-2 Infection. NCT04443881.

[120] ClinicalTrials.gov Anakinra in Adults with Severe COVID-19 and Features of Cytokine Storm Syndrome: A Randomized, Double-Blind, Placebo-Controlled Trial. NCT04603742.

[121] Stebbing J., Krishnan V., de Bono S., Ottaviani S., Casalini G., Richardson P.J., Monteil V., Lauschke V.M., Mirazimi A., Youhanna S. (2020). Mechanism of baricitinib supports artificial intelligence-predicted testing in COVID-19 patients. EMBO Mol. Med..

[122] Ghoreschi K., Laurence A., O’Shea J.J. (2009). Janus kinases in immune cell signaling. Immunol. Rev..

[123] Cantini F., Niccoli L., Matarrese D., Nicastri E., Stobbione P., Goletti D. (2020). Baricitinib therapy in COVID-19: A pilot study on safety and clinical impact. J. Infect..

[124] Praveen D., Puvvada R.C., Aanandhi V.M. (2020). Janus kinase inhibitor baricitinib is not an ideal option for management of COVID-19. Int. J. Antimicrob. Agents.

[125] Kalil A.C., Patterson T.F., Mehta A.K., Tomashek K.M., Wolfe C.R., Ghazaryan V., Marconi V.C., Ruiz-Palacios G.M., Hsieh L., Kline S. (2020). Baricitinib plus Remdesivir for Hospitalized Adults with Covid-19. N. Engl. J. Med..

[126] The U.S. Food and Drug Administration Coronavirus (COVID-19) Update: FDA Authorizes Drug Combination for Treatment of COVID-19. https://www.fda.gov/news-events/press-announcements/coronavirus-covid-19-update-fda-authorizes-drug-combination-treatment-covid-19.

[127] ClinicalTrials.gov A Randomized, Double-Blind, Placebo-Controlled, Parallel-Group Phase 3 Study of Baricitinib in Patients with COVID-19 Infection. NCT04421027.

[128] ClinicalTrials.gov A Phase II Randomized Double-Blind Trial of Baricitinib or Placebo Combined with Antiviral Therapy in Patients with Moderate and Severe COVID-19. NCT04373044.

[129] ClinicalTrials.gov BARICIVID-19 STUDY: MultiCentre, Randomised, Phase IIa Clinical Trial Evaluating Efficacy and Tolerability of Baricitinib as Add-on Treatment of In-Patients with COVID-19 Compared to Standard Therapy. NCT04393051.

[130] ClinicalTrials.gov Treatment of Moderate to Severe Coronavirus Disease (COVID-19) in Hospitalized Patients. NCT04321993.

[131] ClinicalTrials.gov A Multicenter, Adaptive, Randomized Blinded Controlled Trial of the Safety and Efficacy of Investigational Therapeutics for the Treatment of COVID-19 in Hospitalized Adults (ACTT-4). NCT04640168.

[132] Patel S.K., Saikumar G., Rana J., Dhama J., Yatoo M.I., Tiwari R., Rodríguez-Morales A.J., Dhama K. (2020). Dexamethasone: A boon for critically ill COVID-19 patients?. Travel Med. Infect. Dis..

[133] Ahmed M.H., Hassan A. (2020). Dexamethasone for the Treatment of Coronavirus Disease (COVID-19): A Review. SN Compr. Clin. Med..

[134] Lammers T., Sofias A.M., van der Meel R., Schiffelers R., Storm G., Tacke F., Koschmieder S., Brümmendorf T.H., Kiessling F., Metselaar J.M. (2020). Dexamethasone nanomedicines for COVID-19. Nat. Nanotechnol..

[135] Giles A.J., Hutchinson M.-K.N.D., Sonnemann H.M., Jung J., Fecci P.E., Ratnam N.M., Zhang W., Song H., Bailey R., Davis D. (2018). Dexamethasone-induced immunosuppression: Mechanisms and implications for immunotherapy. J. Immunother. Cancer.

[136] (2020). Dexamethasone in Hospitalized Patients with Covid-19. N. Engl. J. Med..

[137] Tomazini B.M., Maia I.S., Cavalcanti A.B., Berwanger O., Rosa R.G., Veiga V.C., Avezum A., Lopes R.D., Bueno F.R., Silva M.V.A.O. (2020). Effect of dexamethasone on days alive and ventilator-free in patients with moderate or severe acute respiratory distress syndrome and COVID-19: The CoDEX randomized clinical trial. JAMA.

[138] Lamontagne F., Agoritsas T., Macdonald H., Leo Y.-S., Diaz J., Agarwal A., Appiah J.A., Arabi Y., Blumberg L., Calfee C.S. (2020). A living WHO guideline on drugs for covid-19. BMJ.

[139] ClinicalTrials.gov Comparison of Efficacy of Dexamethasone and Methylprednisolone in Moderate to Severe Covid 19 Disease. NCT04603729.

[140] ClinicalTrials.gov Effect of Dexamethasone in Patients with ARDS and COVID-19—Prospective, Multi-Centre, Open-Label, Parallel-Group, Randomized Controlled Trial (REMED Trial). NCT04663555.

[141] ClinicalTrials.gov Efficacy of Low or High Dose of Dexamethasone in Patients with Respiratory Failure by COVID-19. NCT04726098.

[142] ClinicalTrials.gov Dexamethasone Combined with Hydroxychloroquine Compared to Hydroxychloroquine Alone for Treatment of Severe Acute Respiratory Distress Syndrome Induced by Coronavirus Disease 19 (COVID-19): A Multicentre, Randomised Controlled Trial. NCT04347980.

[143] ClinicalTrials.gov Randomized Controlled Phase 2/3 Clinical Trial of NA-831 Alone or with Atazanavir, or NA-831 with Dexamethasone, or Atazanavir with Dexamethasone in the Treatment of COVID-19 Infection. NCT04452565.

